# In vitro antimicrobial susceptibility testing of human *Brucella melitensis* isolates from Qatar between 2014 – 2015

**DOI:** 10.1186/s12866-015-0458-9

**Published:** 2015-06-16

**Authors:** Anand Deshmukh, Ferry Hagen, Ola Al Sharabasi, Mariamma Abraham, Godwin Wilson, Sanjay Doiphode, Muna Al Maslamani, Jacques F Meis

**Affiliations:** Microbiology Division, Hamad General Hospital, Doha, Qatar; Department of Medical Microbiology and Infectious Diseases, Canisius-Wilhelmina Hospital, Nijmegen, The Netherlands; Microbiology Division, Al Khor Hospital, Doha, Qatar; Department of Infectious Diseases, Hamad General Hospital, Doha, Qatar; Department of Medical Microbiology, Radboud University Medical Center, Nijmegen, The Netherlands

**Keywords:** Brucella melitensis, Brucellosis, Antimicrobial susceptibility testing, Qatar

## Abstract

**Background:**

Brucellosis is one of the most common zoonotic disease affecting humans and animals and is endemic in many parts of the world including the Gulf Cooperation Council region (GCC). The aim of this study was to identify the species and determine the antimicrobial susceptibility pattern of *Brucella* strains isolated from clinical specimens, from Qatar.

**Results:**

We evaluated 231 *Brucella* isolates. All isolates were identified as *B. melitensis*. All the isolates were susceptible to doxycycline, tetracycline, streptomycin, gentamicin, trimethoprim / sulfamethoxazole and ciprofloxacin except rifampicin, where 48 % of the strains showed elevated MICs (>1 mg/L). The rifampicin-resistance related hotspots within the *rpoB* gene were amplified and sequenced using PCR and no *rpoB* mutations were found in strains with rifampicin MICs of >2 mg/L.

**Conclusion:**

This study identified *B. melitensis* as the etiological agent of brucellosis in Qatar. No resistant isolates were detected among conventionally used antimicrobial agents.

## Background

Brucellosis is a worldwide zoonotic disease in both animals and humans with an estimated 500,000 new cases annually [1]. Although brucellosis is a notifiable disease in many countries, it is probably underreported and official numbers do not reflect the true incidence of this infection [2]. Thus the true incidence of human brucellosis is unknown and the estimated burden of the disease varies widely, from < 0.03 to >160 per 100,000 population. The highest recorded incidence of human brucellosis occurs in the Middle East and Central Asia [3]. Brucellosis is transmitted to humans by direct contact with infected animals or consumption of unpasteurized or inadequately cooked milk or milk products, inhalation of infected aerosolized particles and, to a lesser extent, meat derived from cattle, sheep, goats, pigs, camels, yaks, buffaloes or dogs. Four species of the genus *Brucella* are usually pathogenic for humans, which include *B. melitensis* (from sheep/goats and camel), *B. abortus* (from cattle and other bovine animals), *B. suis* (from pigs), and *B. canis* (from dogs) [4]. Routine susceptibility testing of *Brucella* species is not performed due to the need for containment level 3 facilities [4] and concerns of laboratory-acquired infections [5]. The main objective of this study was to determine the antimicrobial susceptibility pattern of human *Brucella* isolates from patients in Qatar collected over a 10 -year period.

## Methods

### Bacterial isolates

A total of 231 strains of *Brucella* isolates obtained from various clinical specimens (blood n = 225 and synovial fluid n = 6) collected between January 2005 and July 2014 from patients attending Hamad General Hospital and Al Khor Hospital, Qatar, were included in the study. All the isolates were collected as a part of standard patient care. Identification of isolates was based on colony morphology, Gram stain, oxidase, catalase, Vitek 2 compact (bioMerieux, Durham, USA) and Maldi-TOF MS (Bruker Daltonics, Bremen, Germany). For Maldi-TOF MS, cultures were processed using the ethanol/formic acid/acetonitrile protocol [6]. All cultures were processed in a Class II biological safety cabinet in a negative pressure room.

### Antimicrobial susceptibility testing

Susceptibility of seven antibiotics (doxycycline, tetracycline, streptomycin, gentamicin, rifampicin, trimethoprim/sulfamethoxazole and ciprofloxacin) was determined with a gradient strip method (E-test strips, bioMerieux, Marcy L’Etoile, France). The antimicrobial strips were placed on Muller Hinton blood agar according to the manufacturer’s guidelines and read after 48 h incubation in ambient air at 37 °C. Minimum inhibitory concentrations (MIC) breakpoints of streptomycin, gentamicin, tetracycline, doxycycline and trimethoprim-sulfamethoxazole were used as recommended by CLSI [7]. As MIC breakpoints for *Brucella* against rifampicin and ciprofloxacin have not been established, guidelines for slow-growing bacteria (*H.influenzae*) were used [8, 9]. The MIC was interpreted as the value at which the inhibition zone intercepted the scale on the E-test strip and the MIC values were rounded up (to the next higher dilution) with the corresponding MIC of the microbroth dilution method. MIC_50_ and MIC_90_ levels were defined as the lowest concentration of the antibiotic at which 50 % and 90 % of the isolates were inhibited, respectively.

The reference strains- *H. influenza* ATCC 10211, *E.coli* ATCC 25922, *S.pneumoniae* ATCC 49619 and *S.aureus* ATCC 29213 were used as quality control.

### PCR assay of *rpoB* gene

For isolates (n = 17) that showed a rifampicin MIC of > 2 mg/L, the rifampicin-resistance related hotspots within the *rpoB* gene were amplified and sequenced as described before [10]. The sequences were compared to *rpoB* sequences of wildtype and resistant isolates as provided by other studies [10, 11].

## Results

**Fig. 1:**
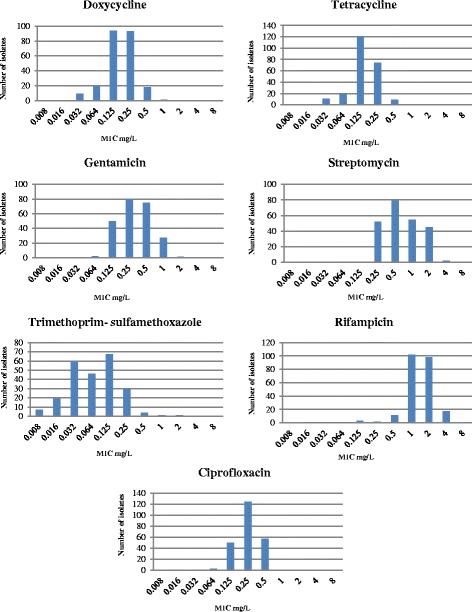
Distribution of MIC values in indicated antimicrobials of *B. melitensis* from Qatar

All the isolates were identified by Vitek II compact and Maldi-TOF as *B. melitensis.* Table 1 represents MIC range; MIC_50_ and MIC_90_ of each antibiotic tested and MIC distributions are shown in Fig. 1.

**Table 1:** Antimicrobial susceptibility of *B.melitensis*

	Range (mg/L)	MIC_50_ (mg/L)	MIC_90_ (mg/L)	Breakpoints for susceptibility (mg/L)
Doxycycline	0.032–1	0.125	0.25	≤1 [7]
Tetracycline	0.032–0.5	0.125	0.25	≤ 1 [7]
Gentamicin	0.064–2	0.25	0.5	≤ 4 [7]
Streptomycin	0.125–4	0.5	2	≤ 8 [7]
Trimethoprim-Sulfamethoxazole	0.008–2	0.064	0.25	≤ 2/38 [7]
Rifampicin	0.008–4	1	2	≤ 1 [8]
Ciprofloxacin	0.064–0.5	0.25	0.5	≤ 1 [8]

All the isolates were susceptible to doxycycline (MIC_90_, 0.25 mg/L), gentamicin (MIC_90_, 0.5 mg/L), streptomycin (MIC_90_, 2 mg/L), trimethoprim-sulfamethoxazole (MIC_90_, 0.25 mg/L) and tetracycline (MIC_90_, 0.25 mg/L). In addition, ciprofloxacin (MIC_90_, 0.5 mg/L) showed 100 % susceptibility when MIC breakpoint criteria for *H.influenzae* [8] were used. For rifampicin, the MIC values ranged from 0.008 to 4 mg/L and when compared with MIC breakpoint criteria for *H.influenzae* [8], resistance (MIC ≥ 2 mg/L) was demonstrated in 48 % strains. After sequencing of the rpoB gene, no mutations were found in isolates with MIC of > 2 mg/L (n = 17).

## Discussion

*Brucella melitensis* is the commonest etiological agent causing human brucellosis worldwide. Intracellular localization of *Brucella* species within monocytes and macrophages of the reticulo-endothelial system limits the choices of antimicrobial agents for effective treatment of systemic and localized brucellosis. Although a variety of antimicrobial agents appear to be active in vitro, the results of susceptibility testing do not always correlate with clinical efficacy [4].

The recommended method for antimicrobial susceptibility testing of *Brucella* species is microbroth dilution using unsupplemented *Brucella* broth [7]. When the E-test method was compared with microbroth dilution, Gur et al. found no significant variations in obtained MIC values [12]. This method appear to be reliable, reproducible, easily performed, and produces similar results to those of conventional methods for *Brucella* on Muller Hinton blood agar [13, 14] and it is therefore that we used E-test for susceptibility testing.

Amongst tetracyclines, doxycycline is the most common antimicrobial drug used for treatment of uncomplicated brucellosis in adults and children of more than 8 years [4]. In the present study, MICs of tetracycline (range - 0.032–0.5 mg/L) and doxycycline (range - 0.032–1 mg/L) are found to be in the susceptible category and MIC_50_ and MIC_90_ values are consistent with previous reports [9, 13, 15, 16]. However, increased MICs of doxycycline have been reported in a study from Mexico [17].

The World Health Organization (WHO) recommends doxycycline (6 weeks) in combination with aminoglycosides (streptomycin or gentamicin for 2–3 weeks) or rifampicin (6 weeks) [4]. The most preferred and effective aminoglycoside in treatment of brucellosis is streptomycin [4, 18, 19]. Combination with streptomycin was found to be superior as compared to rifampicin, in terms of relapse or treatment failure [18]. Even though gentamicin is more active in vitro and is associated with fewer side effects than streptomycin, there are not enough studies, which justify the replacement of streptomycin [4, 19]. In the present study, all the strains were susceptible to streptomycin (0.125–4 mg/L) and gentamicin (0.064–2 mg/L) and found to be in the range described previously [9, 15]. Nevertheless, a high MIC_90_ of streptomycin (8 mg/L) was reported from Turkey [16].

Another commonly used drug in treatment of brucellosis in combination with doxycycline is rifampicin. In spite of the proven efficacy with aminoglycoside combination, due to the need for parenteral administration, convenience and poor patient compliance, healthcare professionals prefer the all-oral regimen of doxycycline/rifampicin [18, 20]. A meta-analysis on various combinations used for the treatment of brucellosis, has shown higher relapse rate with doxycycline/rifampicin when compared to doxycycline/streptomycin regimen [21]. It has been suggested that rifampicin may enhance the plasma clearance of doxycycline resulting in lower doxycyline levels [22] and the possibility that its use could contribute to emergence of rifampicin- resistant *Mycobacterium tuberculosis* in endemic countries [23]. For in-vitro susceptibility testing, MIC breakpoints of rifampicin against *Brucella* has not been established and therefore these organisms cannot be confidently characterised as susceptible, intermediate or resistant. However, when compared with CLSI reference values for *H.influenzae* [8], in the present study, 48 % of *Brucella* strains showed elevated MIC’s of rifampicin (>1 mg/L). Higher MIC’s to rifampicin has been reported previously from Egypt (64 %) [9] and Malaysia (70 %) [15]. However, the impact of high MIC’s on clinical outcome in these countries is not known. In Qatar, in a retrospective cohort study, combination of doxycyline with streptomycin was found to be the preferred regimen followed by doxycyline with rifampicin and no relapse or therapeutic failures were detected [24].

Mutations conferring rifampin resistance are confined almost exclusively to the *rpoB* gene in most organisms and result in a decreased affinity of the DNA-dependent RNA polymerase to the antibiotic. Alternative mechanisms of rifampin resistance also have been observed [25]. Along with mutations in the *rpoB* gene, excitation of several metabolic processes may also be a contributing factor in conferring rifampicin resistant in *Brucella* [26]. We did not find any mutations in the *rpoB* gene in isolates with elevated MIC’s in this study, findings consistent with previous studies [14, 27, 11]. However, further research is required to find possible other mechanisms of rifampicin resistance in *Brucella.*

Trimethoprim/sulfamethoxazole (TMP/SMX) is an alternative agent recommended for treatment of brucellosis in pregnancy and in children under 8 years old. It has also been recommended for treatment of osteo-articular complications, neurobrucellosis and endocarditis, in combination with doxycycline, aminoglycoside and rifampicin [4]. TMP/SMX, when used in combination with rifampicin, was found to be effective in pediatric patients (8 weeks regimen). High relapse rates were noted when TMP/SMX was used as a monotherapy in pediatric brucellosis [28]. Development of resistance to TMP/SMX to *B. melitensis* is an important issue. High rate of resistance to TMP/SMX has been reported from previous studies [17, 29]. In the present study, TMP/SMX was found to be susceptible with low MIC_50_ (0.064 mg/L) and MIC_90_ (0.25 mg/L) when compared to other antimicrobials which is consistent with previous reports [9, 13, 15].

Ciprofloxacin is a potential alternative therapeutic option in the treatment of brucellosis with excellent oral bioavailability and reaching high concentrations in phagocytic cells [30]. However, ciprofloxacin is not recommended for monotherapy due to the lack of bactericidal activity at the intracellular acidic pH [31], high rate of relapse and the risk of development of overall fluoroquinolone resistance in the community [32]. Therefore, its use in combination with doxycycline is recommended as an acceptable alternative but not as first line regimen [29, 33]. We found ciprofloxacin MICs (0.064–0.5 mg/L) in the susceptible range when compared to MIC breakpoint criteria of slow growing bacteria and these findings are consistent with previous studies [9, 16]. Ciprofloxacin based therapy might play a role as an alternative regimen especially in patients with intolerance to commonly used drugs and relapsed disease [30].

## Conclusion

In summary, this study identified *B. melitensis* as the etiological agent of brucellosis in Qatar. No resistant isolates were detected among conventional antimicrobial agents. Periodic monitoring of antimicrobial resistance in the Middle East, especially in the Gulf Cooperating Council (GCC) in light of the emerging resistance and development of CLSI or EUCAST interpretive criteria for rifampicin are needed.
